# Potential effect of Sildenafil beyond pulmonary hypertension in a patient with diffuse systemic sclerosis and cryoglobulinemic vasculitis

**DOI:** 10.1186/2193-1801-3-559

**Published:** 2014-09-26

**Authors:** Tamer A Gheita, Hussam Ammar, Sanaa A Kenawy

**Affiliations:** Rheumatology and Clinical Immunology, Faculty of Medicine, Cairo University, Cairo, Egypt; Internal Medicine, University of Texas Health Science Center, Houston, USA; Pharmacology, Royal College of Surgeons, London, UK; Pharmacology and Toxicology Department, Faculty of Pharmacy, Cairo University, Cairo, Egypt

**Keywords:** Diffuse systemic sclerosis, Pulmonary hypertension, Cryoglobulinemic vasculitis, Sildenafil

## Abstract

**Introduction:**

Pulmonary arterial hypertension (PAH) is a serious complication of systemic sclerosis (SSc), has a dramatic impact on prognosis and survival and is a leading cause of death.

**Case description:**

A 40 years old female patient with difuse cutaneous SSc (dcSSc) presented with progressive dyspnea, choking sensation, cough, abdominal distension, constipation and dysphagia to solids. The muscle power was mildly reduced and multiple purpuric eruptions were present on the legs of variable sizes. The patient was ANCA negative and had positive cryoglobulinemia. The hepatitis C virus test was positive and the skin biopsy histopathology proved small vessel (leucocytoclastic) vasculitis. The modified Rodnan total skin score (MRSS) was 37. There was deterioration of the pulmonary function tests and transesophageal echocardiography revealed PAH (RVSP 60 mmHg). Sildenafil 50 mg/day resulted in a remarkable improvement of the dyspnea and Raynauds’ with a significant improvement of the skin tightness as the MRSS became 22. The small vessel vasculitic rash remarkably improved and the RVSP became 34 mmHg with a dramatic improvement of the PAH.

**Discussion and evaluation:**

Sildenafil enhances vasodilatation, has antiproliferative effects and is effective in the treatment of PAH. The remarkable improvement in the vasculitic skin lesions in this case after sildenafil is the second report after the described dramatic improvement of small vessel vasculitis in a case with Takayasu arteritis. The emerging trends make it necessary to exploit the full therapeutic potential of Sildenafil in scleroderma and PAH with other extrapulmonary manifestations.

**Conclusion:**

We report a very rare association of dcSSc with small vessel cryoglobulinemic vasculitis with a remarkable improvement after sildenafil.

## Case presentation

A 40 year-old female with diffuse cutaneous systemic sclerosis (dcSSc) for 3 years was admitted with palpitations (24 hour Holter monitor showed ventricular ectopics). The patient was started on deltiazem 40 mg tid. She developed progressive dyspnea, chocking sensation and cough. The patient started to have abdominal distension, constipation and dysphagia to solids. Arthritis of the knee started to occur with puffiness of the hands and 10 minute morning stiffness and eventually she was unable to close a fist (Her sister has rheumatoid arthritis). She started to feel proximal weakness on going upstairs (grade 4 muscle power). The menstrual cycle became irregular and there would be urgency of urine. Laboratory investigations of the patient are shown in Table 1. The case study conforms to the 1995 Helsinki declaration and the patients gave an informed consent.

**Figure 1:**
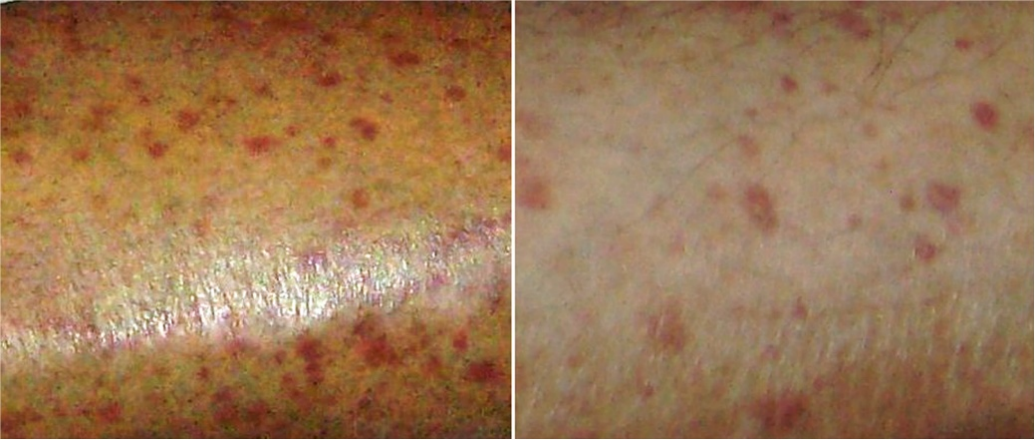
**Purpuric skin rash (small vessel vasculitis) in a 40 year old female with diffuse cutaneous systemic sclerosis (dcSSc) and pulmonary arterial hypertension (PAH) before (left) and after (right) treatment with sildenafil.**

There was pitting edema of extremities, peripheral skin tightness, coldness and Raynauds with sluggish peripheral circulation. Skin tightness was increased as reflected by the increased modified Rodnan total skin score (MRSS) which was 37. Multiple purpuric eruptions were present on the legs and of variable sizes (Figure 1). Skin biopsy histopathology proved small vessel (leucocytoclastic) vasculitis of the purpuric rash. The patient had a positive hepatitis C virus (HCV) test by PCR and positive cryoglobulinemia. The pulmonary function test was consistent with a restrictive pattern with progressive deterioration (FEV1 (58.2%), FVC (64.7%), DLCO (16.9%) and FEV1/FVC (74.5%) compared to the results of her preceding follow-up. Transesophageal echocardiography revealed pulmonary hypertension (RVSP 60 mmHg), EF (63%) with moderate pericardial effusion which were verified by a non-contrast CT chest.

**Table 1:** **Laboratory features of the diffuse cutaneous systemic sclerosis patients with pulmonary hypertension and small vessel (cryoglobulinemic) vasculitic rash**

Laboratory investigations	
Hb (g/dl)	15.6
WBC (×10^3^/mm^3^)	5.6
Platelets (×10^3^/mm^3^)	246
CK (U/L)	81
LDH (U/L)	691
ESR (mm/1^st^ hr)	114
Sodium (mmol/L)	142
Potassium (mmol/L)	3.8
Calcium (mg/dl)	9.2
Phosphorus (mg/dl)	3.3
PT (seconds)	17
INR	1.4
PTT (seconds)	34.3
Triglycerides (mg/dl)	102
Cholesterol (mg/dl)	127
Cholesterol/HDL	4.4
LDL/HDL	2.7
AST (U/L)	19
ALT (U/L)	7
ALP (IU/L)	59
Creatinine (mg/dl)	0.6
Urea (mg/dl)	9
SUA (mg/dl)	6.4
RF	Negative
ANA	Positive (1:40) homogeneous
Anti-ds DNA	Negative
Anti-Scl-70	Positive
Anticentromere	Negative
U1RNP	negative
Anti-Ro (U/ml)	Positive (28.2)
Anti-La (U/ml)	Positive (17.9)
ANCA	Negative
Cryoglobulinemia	Positive
HCV (PCR)	Positive

Hb: Hemoglobin, WBC: White blood cells count, CK: creatine kinase, LDH: lactate dehydrogenase, ESR: Erythrocyte sedimentation rate, PT: prothrombin time, INR: Inverse neutralization ratio, PTT: partial thromboplastin time, LDL: low density lipoprotein, HDL: high density lipoprotein, AST: Aspartate transaminase, ALT: alanine transaminase, SUA: serum uric acid, RBF: rheumatoid factor, ANA: antinuclear antibody, ds DNA: double stranded deoxyribonucleic acid, Scl-70: scleroderma 70, U1RNP: Uridine 1 ribonucleoprotein, ANCA: antineutrophil cytoplasmic antibody, HCV: hepatitis C virus.

The patient was prescribed prednisolone 40 mg/day, warfarin 3 mg/day, furosemide 20 mg with spironolactone 100 mg, verapamil 120 mg/day. In addition, the patient received a daily dose of omeprazole 40 mg, domperidone 10 mg before the main meal, simethicone chew tablets, calcium carbonate 500 mg and colchicines 0.5 mg twice daily for a month. There was slight improvement of the dyspnea and skin rash while all other symptoms persisted. The ESR was reduced from 114 to be 85 mm/1^st^ hr.

One week later it was decided to add *sildenafil* 50 mg/day with a remarkable improvement of the dyspnea and Raynauds’ with a reduction of the peripheral edema as well as a significant improvement of the skin tightness as the MRSS became 22. The small vessel vasculitic rash remarkably improved (Figure). The pulmonary function tests significantly improved: FEV1 77%, FVC 79.2%, DLCO 45.2%, FEV1/FVC 88.6. The echocardiography results were enhanced with an EF 73% and a reduction of the pericardial effusion. The RVSP became 34 mmHg with a dramatic improvement of the pulmonary hypertension. The patient significantly improved with a favorable clinical and functional outcome on the regular follow ups for the following year.

Using a selective pulmonary vasodilator as sildenafil contributes to the significant improvement of the clinical conditions and pulmonary hemodynamics which benefits patients with severe pulmonary hypertension resistant to conventional therapy (Catapano-Minotti et al. 2008; Shahin 2006). The pulmonary arterial hypertension associated with connective tissue disease is difficult to manage, and has a poor prognosis. The PDE5 inhibitor ‘sildenafil’ enhances vasodilatation, has antiproliferative effects, and is effective in the treatment of PAH (Badesch et al. 2007). The vasculopathy associated with SSc is considered noninflammatory, yet frank vasculitis can complicate it posing diagnostic and therapeutic challenges (Kao and Weyand 2010).

We report a very rare association of dcSSc with small vessel cryoglobulinemic vasculitis. It has been reported that cryoglobulinemic vasculitis is rarely encountered in SSc patients (Kao and Weyand 2010). The remarkable improvement in the vasculitic skin lesions in this case after sildenafil is the second report after the described dramatic improvement of small vessel vasculitis in a case with Takayasu arteritis (Uthman and Chaaban 2006). The emerging trends make it necessary to exploit the full therapeutic potential of this class of drugs ‘sildenafil’ in scleroderma and PAH with other extrapulmonary manifestations.
